# Editorial: COVID-19 crisis creates opportunity towards global monitoring & surveillance

**DOI:** 10.3389/fcimb.2025.1601046

**Published:** 2025-06-19

**Authors:** Yasir Waheed, Zobia Noreen, Aftab Ahmad, Habib Bokhari

**Affiliations:** ^1^ NUST School of Health Sciences, National University of Sciences and Technology (NUST), Islamabad, Pakistan; ^2^ Operational Research Center in Healthcare, Near East University, Nicosia, Cyprus; ^3^ University of Economics and Human Sciences in Warsaw, Warsaw, Poland; ^4^ Department of Biosciences, COMSATS University Islamabad, Islamabad, Pakistan; ^5^ Department of Microbiology, Kohsar University, Murree, Pakistan

**Keywords:** COVID-19, monitoring, surveillance, wastewater, transmission

COVID-19 (Coronavirus Disease 2019) is caused by the SARS-CoV-2 virus, a novel coronavirus strain that emerged in late 2019. It’s first case was reported in late 2019 and rapidly spread worldwide, leading to a global pandemic. The disease is mainly transmitted from person to person through droplets when the infected person coughs, sneezes, or even talks (Stadnytskyi et al., 2021). The virus is also transmitted in aerosols and fomite. Over 6.8 million people died of COVID-19 (Worldometer), and a significant number of individuals who survived the infection experience long COVID.

In the Research Topic entitled “*COVID-19 crisis creates opportunity towards global monitoring & surveillance*” we gathered 6 manuscripts including 4 original articles, 1 review article and 1 mini review.

Sun et al., developed a mortality prediction model among severely ill patients with COVID-19 in study entitled “*Development and validation of a prediction model for mortality in critically ill COVID-19 patients*”. The study included 137 patients from the ICU during April-June 2022. Two disease prediction models were developed in this study. The first model comprises seven variables chosen by the LASSO regression, and the second model comprises three independent variables. The LASSO regression-based model can accurately predict outcomes among critically ill patients with COVID-19 and justify early intervention. External validation via large-scale prospective studies is recommended.

The surveillance of viruses in sewerage water provides a good indicator of the circulation of viral pathogens in a population. Maryam et al., published a review article entitled “*COVID-19 surveillance in wastewater: An epidemiological tool for the monitoring of SARS-CoV-2*”, in which they stressed the monitoring and early surveillance of viral RNA from wastewater. SARS-CoV-2 RNA has been found in ground water, wastewater, sludge, and surface water during the COVID-19 period. Additionally, SARS-CoV-2 RNA was detected in natural waterways from unsanitary locations. The detection of viral RNA in wastewater is also limited by several factors, such as the possibility of genome stability in wastewater, low viral RNA concentrations, and ineffective viral concentration techniques.

SARS-CoV-2 is a highly infectious virus that can be transmitted via airway instrumentation, particularly in otolaryngology and intensive medicine. Albaharna et al., published research entitled “*Efficacy of chlorin dioxide wipes in disinfecting airway devices contaminated with Covid-19*”. The study was conducted on 38 samples from 19 subjects (scopes). Only scopes with a positive COVID-19 swab before disinfection were tested. The post-disinfection swabs were negative for COVID-19 after disinfection using chlorine dioxide wipes. Chlorine dioxide wipes were identified as a quick, convenient, and effective option for disinfecting COVID-19-contaminated airway endoscopes.

The clinical profile of patients with COVID-19 is important in determining patient outcomes. Geng et al., published a study entitled “*Understanding clinical characteristics influencing adverse outcomes of Omicron infection: a retrospective study with propensity score matching from a Fangcang hospital*”. This study showed that older patients develop severe COVID-19 have more symptoms and comorbidities. Fever and diarrhea were identified as risk factors for severe disease.

In this Research Topic Mu et al., published a study entitled “Implications of LDH in patients with coronavirus disease 2019 pneumonia”. The study included 101 patients who presented with COVID-19 pneumonia. Patients were divided into groups according to disease severity and recovery after treatment. The findings indicated that patients with severe pneumonia had higher serum lactic dehydrogenase (LDH) and neutrophil counts than those with common pneumonia. According to the study findings, LDH levels play a crucial role in determining the severity and outlook of COVID-19 pneumonia.

In this Research Topic Yang et al., published a mini review entitled “*Changing patterns of infectious diseases in children during the COVID-19 pandemic*”, in which they discussed the patterns of different infectious diseases during the early and late phases of the COVID-19 pandemic. During the initial phase of COVID-19, studies from different countries showed that more than half of children with COVID-19 require hospital admission, with 4% of children requiring mechanical ventilation. This disease pattern changed by 2021, when most children showed mild (58%) or asymptomatic (36%) infections. Strict public health measures, such as mask wearing, curfews, social distancing and closure of childcare facilities and schools, were implemented at the onset of the COVID-19 pandemic, which resulted in a decline in different viral and bacterial infections in children. This lack of exposure has raised concerns about a “debt” in their immunity, making individuals more vulnerable to infectious diseases. The “upsurge”, “see-saw,” and “off-season” patterns of several infectious illnesses in children are caused by this immune debt.

In conclusion, fever and diarrhea were identified as risk factors for severe coronavirus disease, and high serum lactic dehydrogenase and neutrophil counts were associated with severe COVID-19 pneumonia. The LASSO regression-based model accurately predicted outcomes among critically ill patients with COVID-19. Surveillance of SARS-CoV-2 RNA in wastewater is an epidemiological tool for monitoring COVID-19 in the population. An efficient solution for cleaning COVID-19-contaminated airway endoscopes is using chlorine dioxide wipes. The immunity debt during the early COVID-19 pandemic led to an upsurge of different infectious diseases among children. We are thankful to the authors and reviewers who contributed to the success of this Research Topic.
